# The Simultaneous Compression of the Median and Ulnar Nerves by Lipoma: A Case Report and Review of the Literature

**DOI:** 10.7759/cureus.59340

**Published:** 2024-04-30

**Authors:** Mohamed Faouzi Hamdi, Hichem Msek

**Affiliations:** 1 Plastic Surgery Unit, Fattouma Bourguiba University Hospital, Monastir, TUN

**Keywords:** nerve compression syndrome, entrapment syndrome, ulnar nerve, median nerve, hand lipoma

## Abstract

Entrapment syndromes in the hand are very common and usually idiopathic. However, it can be the expression of nerve compression by a nearby structure or tumor. Space-occupying lesions are widely reported as a cause of median or ulnar nerve compression in the wrist. Nevertheless, a simultaneous compression of the median and ulnar nerves by a tumor is a rare condition. To the best of our knowledge, only three cases are described in the English literature. Herein, we present a case of a simultaneous compression in the wrist of the median and ulnar nerves by lipoma in a 79-year-old patient while also elucidating the reasons for the rarity of this condition and reviewing its clinical and therapeutic particularities.

## Introduction

Median nerve compression at the wrist is a common condition. It is often idiopathic, stemming from inflammation secondary to microtrauma and repetitive movements. Conversely, ulnar nerve compression at this level is much less frequent. Although compression of either the median or ulnar nerve by a tumor, notably lipomas, is relatively uncommon, it is well documented in the literature. However, simultaneous compression of both nerves by a lipoma remains exceptional. In this paper, we describe a 79-year-old patient with a lipoma compressing both the ulnar and median nerves at the wrist.

## Case presentation

**Figure 1:**
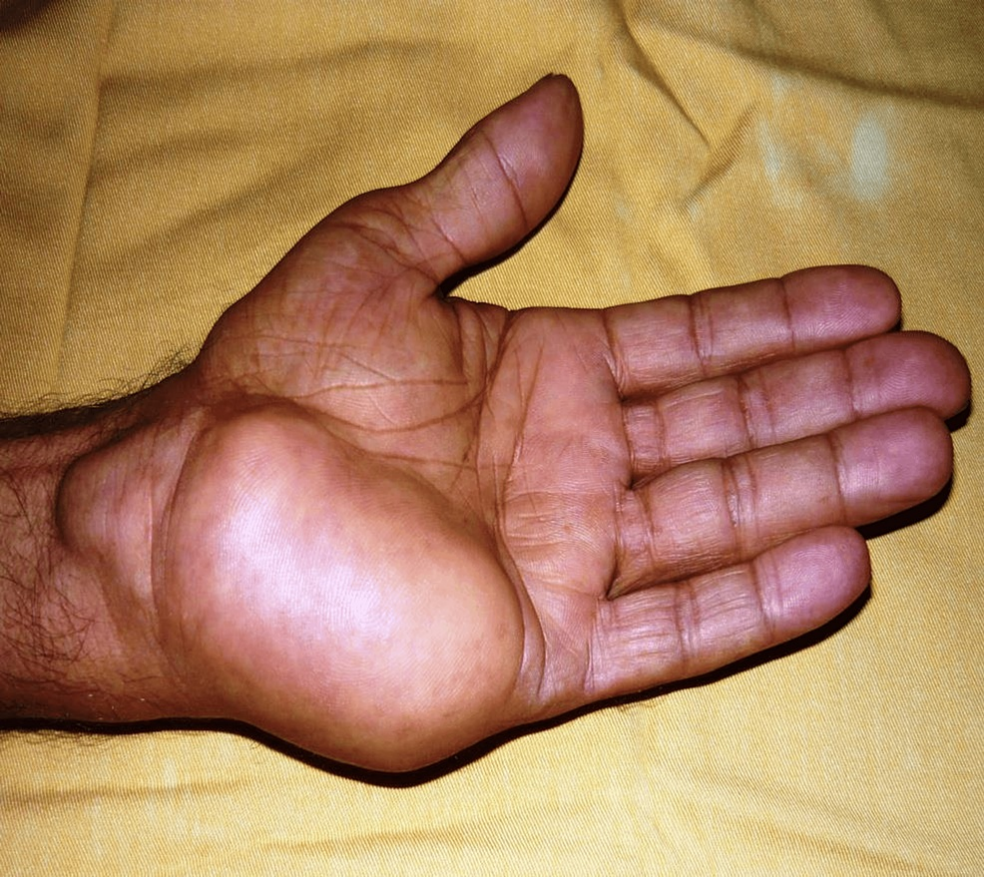
Clinical view of the hand lipoma occupying the hypothenar eminence and extended to the volar aspect of the wrist.

A 79-year-old, right-handed, diabetic male was referred to our outpatient clinic with a complaint of a left hypothenar mass that had been slowly progressing for 29 years. He also reported experiencing paresthesia in the middle and ring fingers for the past four years. During the physical examination, a painless 10 cm-sized mass was noted, located in the left hypothenar region and extending to the volar aspect of the wrist. There was neither inflammation nor intrinsic amyotrophy (Figure 1). Mobility was normal, but grasp was hampered by the tumor. Neurological examination found a sensorial deficit of the middle and ring fingers without motor abnormality. The electromyography (EMG) confirmed the sensory deficit of the median and ulnar nerves.

**Figure 2:**
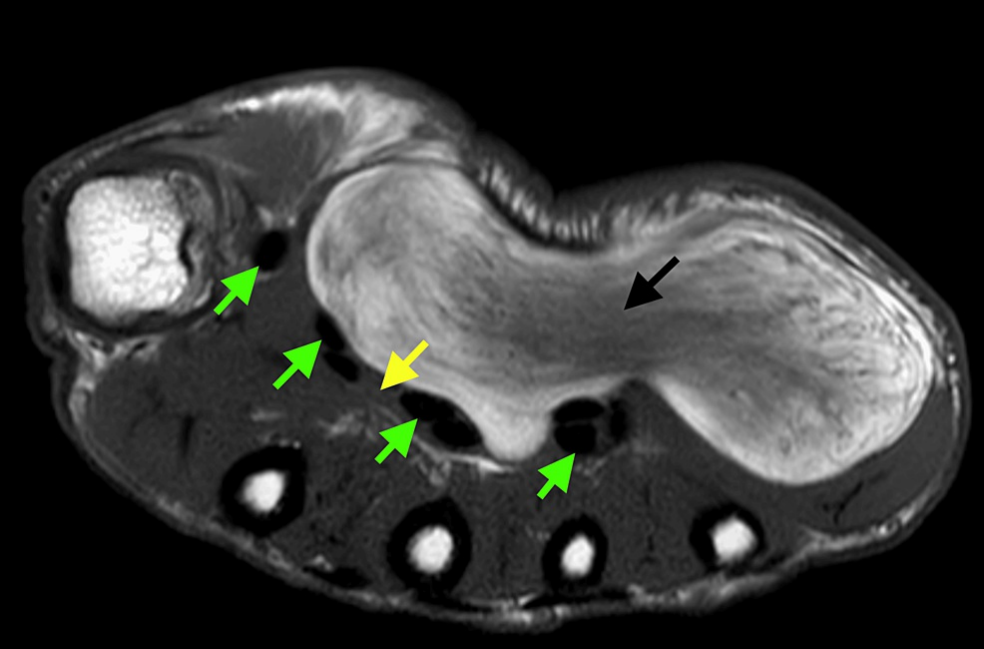
T2-weighted MRI image showing the anatomical relation of the tumor with deep structures of the hand. Black arrow, lipoma; yellow arrow, median nerve; green arrows, flexor tendons. MRI, magnetic resonance imaging

Ultrasound (US) exploration showed a lipomatous tumor of the superficial layer of the hypothenar region. Magnetic resonance imaging (MRI) identified a lipomatous mass of 90 mm x 65 mm x 26 mm located in the left hypothenar region reaching the carpal tunnel where it represses the flexor tendons of the digits and the median nerve (Figure 2).

**Figure 3:**
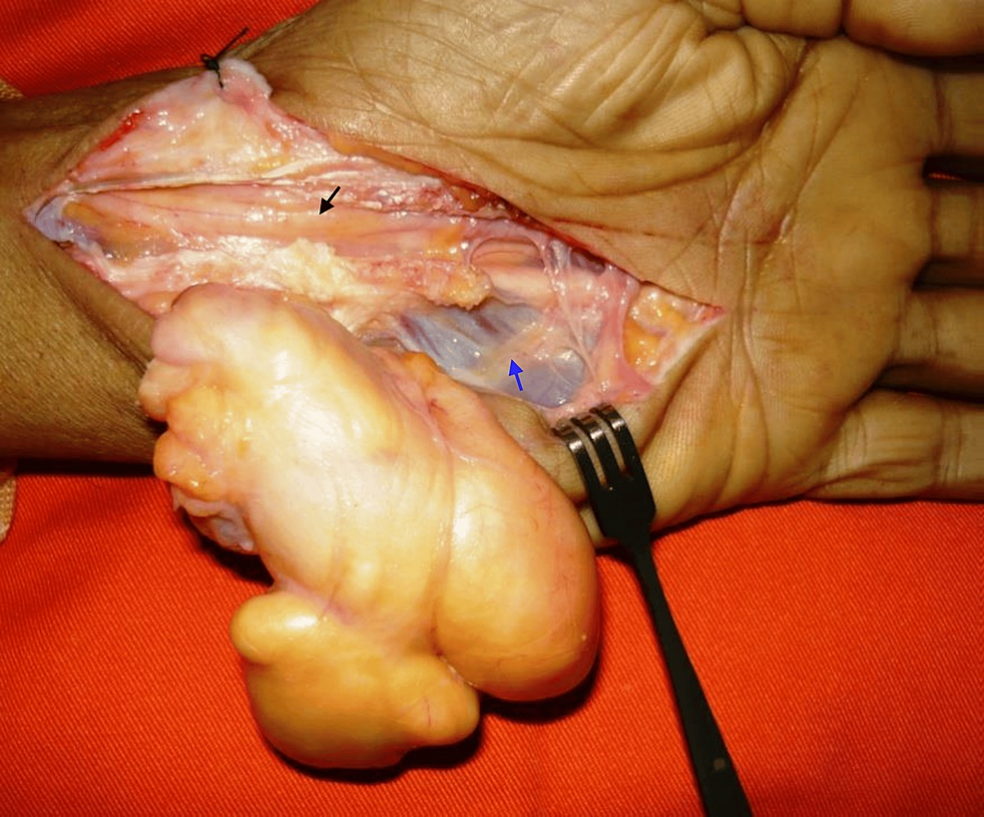
Intraoperative view showing the anatomical relations of the tumor. Black arrow, median nerve; blue arrow, ulnar nerve.

**Figure 4:**
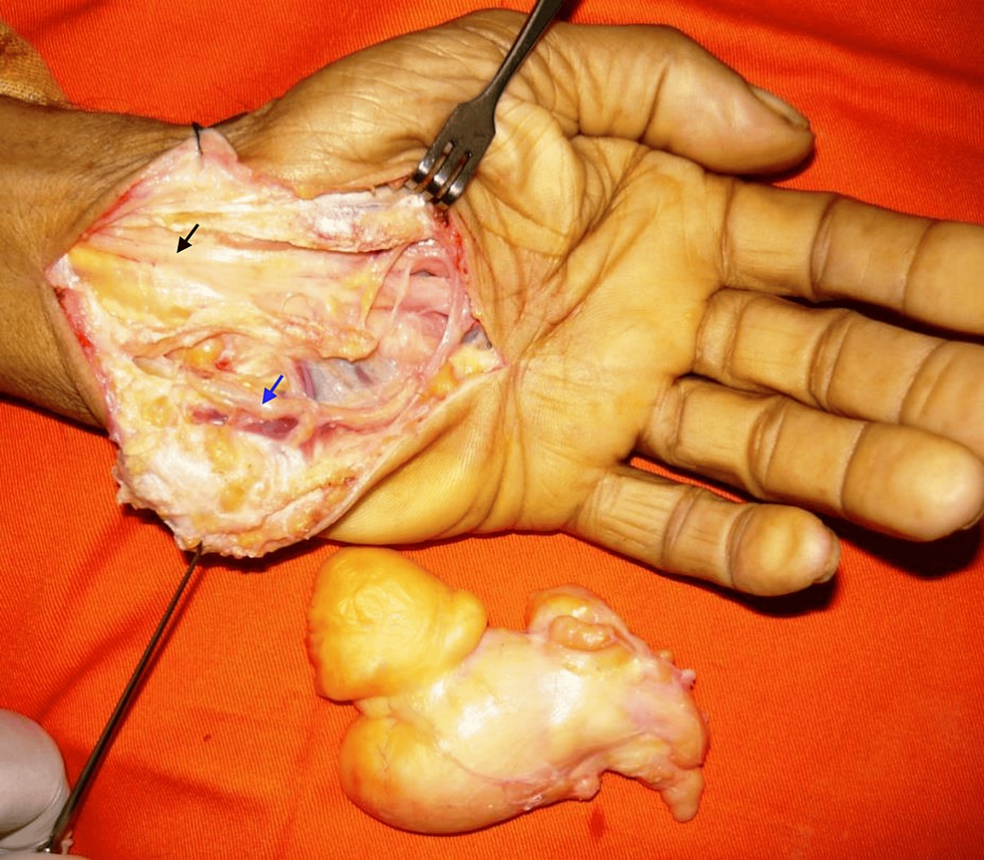
Intraoperative view of tumor resection while preserving the integrity of the median and ulnar nerves. Black arrow, median nerve; blue arrow, ulnar nerve.

The patient underwent surgery under axillary block anesthesia. The lipoma was exposed by a large carpal tunnel incision. The tumor was polylobate, compressing the median nerve and the superficial branch of the ulnar nerve, which were carefully dissected. The lipomatous mass was marginally excised (Figures 3-4). The diagnosis of lipoma was confirmed by histological examination. At one-month follow-up, the patient had a full recovery. At 69-month follow-up, there was no recurrence.

## Discussion

The simultaneous compression of both the median and ulnar nerves by a lipoma is extremely rare. In addition to their report, Unal et al. [1] found only three cases reported in the English literature. If we exclude the case described by Galeano et al. [2] in 2001 where the diagnosis of median nerve compression was made only by EMG and the patient did not have any ulnar nerve compression symptoms, there have been only three reported cases of simultaneous median and ulnar nerve compression by lipoma in the English and French literature (Table 1) [1,3,4].

**Table 1:** Previously reported cases of simultaneous median and ulnar nerve compression by lipoma in the current literature. F, female; M, male; L, left; NS, neurological symptoms; Mo, motor; S, sensorial; EMG, electromyography; US, ultrasound; MRI, magnetic resonance imaging

Case	Author	Age (years)	Sex	Side	Symptoms	Deficit				Exploration	Treatment	Follow-up	Recovery	Recurrence
						Median		Ulnar						
						Mo	S	Mo	S					
1	Pagonis et al. [3], 2011	63	F	L	NS + mass syndrome	+	+	+	+	EMG, X-ray, MRI	Marginal excision	30 months	Complete	No
2	Kamath et al. [4], 2016	49	M	L	NS	-	+	-	+	EMG, MRI	Marginal excision	6 months	Complete	No
3	Unal et al. [1], 2017	50	F	L	NS + mass syndrome	-	+	-	+	EMG, X-ray, MRI	Marginal excision	9 months	Complete	No
4	This study, 2022	79	M	L	NS + mass syndrome	-	+	-	+	X-ray, US, MRI	Marginal excision	69 months	Complete	No

The rarity of the simultaneous compression of the median and ulnar nerves can be explained by the early excision of the tumor. Deep lipomas occurring in the carpal tunnel, which is an inextensible channel, become rapidly symptomatic by compressing the median nerve, and patients are usually treated in this stage. In addition, these tumors tend to expand through the carpal tunnel without reaching the ulnar nerve. On the other hand, superficial lipomas, particularly in the hypothenar eminence, are diagnosed and treated early due to the evident mass syndrome, before reaching the median nerve. Therefore, we think that only neglected superficial lipomas of the hypothenar eminence reaching giant sizes can simultaneously compress the ulnar and median nerves.

Although ulnar nerve compression may be associated with carpal tunnel syndrome in 39.3% of cases [5,6], a simultaneous compression of the median and ulnar nerve by a space-occupying lesion should be suspected whenever symptoms are unilateral with sudden onset [7]. Careful examination can objectify the mass syndrome, which may hide the hypothenar amyotrophy [1,7]. However, the clinical table may be summarized in neurological symptoms [1].

While the clinical examination is sufficient for the diagnosis of idiopathic neural compression, paraclinical exams are needed to explore the tumor's nature and relations. Pagonis et al. [3] noted that preoperative assessment ensures better surgery outcomes.

EMG shows nonspecific signs as an increased latency of nerve conduction [1]. Barreira et al. [7] considered that EMG may be useful for locating and evaluating the compression severity. X-ray shows a radiolucent silhouette of large lipomas [1], calcifications, or bone lesions [7]. Sbai et al. [8] proposed preoperative US exploration to detect space-occupying lesions suspected by clinical examination and encouraged its indication due to its low cost and availability. US describes the nerve anatomy, determines the tumor characteristics, and evaluates the severity of the nerve compression [7]. MRI is the best exploration method for soft tissue tumors. Capelastegui et al. [9] concluded that MRI specificity for soft tissue tumors reaches 94%. Furthermore, for Ozcanli et al. [10], 100% of lipomas found in surgery were correctly diagnosed by preoperative MRI. 

The treatment consists of a complete resection of the tumor. Simple release of nerves is not indicated [7,8]. A carpal tunnel incision is generally sufficient to excise the whole lipoma and carefully release the nerves. The incision can be enlarged in need by a modified Bruner incision [1]. Mini incision and endoscopic techniques are avoided due to the risk of misdiagnosing the mass [7].

Sbai et al. [8] noted a total disappearance of pain and a full-function resumption after lipoma resection. The local recurrence after lipoma surgery is rare [7,8]. Kim et al. [11] estimated its rate to be 5% and noted that deep space lipoma has more chance of recurrence. Barreira et al. [7] explained the recurrence by either incomplete resection or misdiagnosed local causes.

## Conclusions

Simultaneous compression of the median and ulnar nerves is extremely rare. Usually, a lipoma is excised early once clinical expression starts. Only a neglected tumor may grow to reach both the median and ulnar nerves. Diagnosis is suspected when the symptoms are unilateral with a sudden onset. Preoperative assessment is needed, and paraclinical exams ensure good results. The excision of the whole tumor allows nerve decompression and prevents recurrence.
